# Supplementary material for: Identification of photoperiod-induced specific miRNAs in the adrenal glands of Sunite sheep (Ovis aries)
Source: Front Vet Sci. 2022 Jul 22;9:888207. doi: 10.3389/fvets.2022.888207 (PMC9354845; doi:10.3389/fvets.2022.888207)
Supplement: Supplementary file 1 [file Data_Sheet_1.ZIP › Supplementary file/miRDeep2 file.zip!/miRDeep2 file/Know_Structure/SPLP42A2/oar-mir-379.pdf]

miRBase precursor : oar-mir-379
Total read count : 48254
oar-miR-379-5p read count : 45797
oar-miR-379-3p read count : 2437
remaining reads : 20

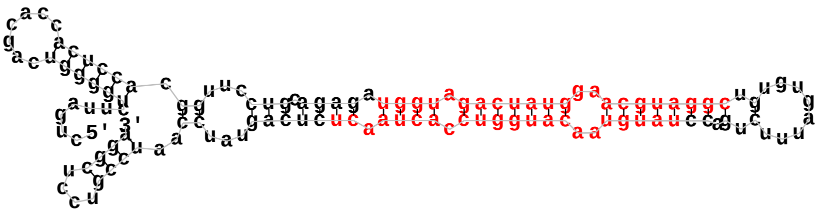

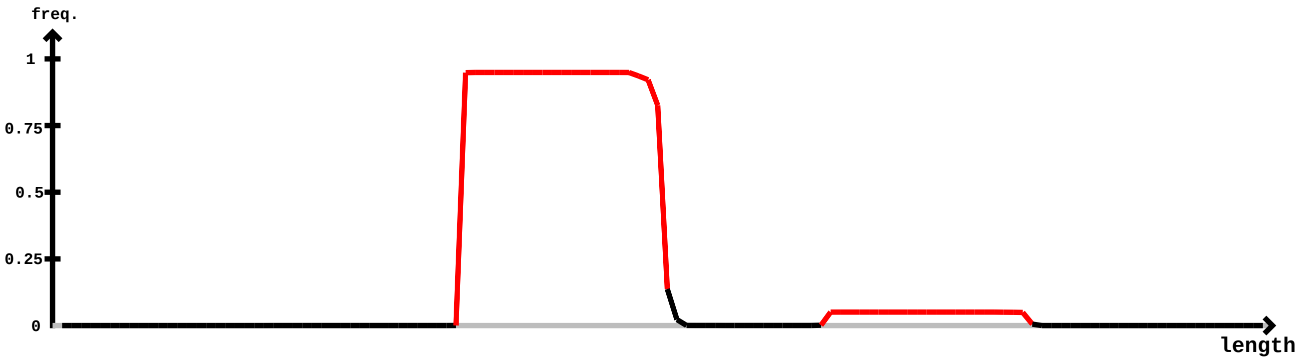

oar-miR-379-5p oar-miR-379-3p

```
5'- cugauucuggggucagcaccacuccacgguuccugcagagaugguagacuauggaacguaggcugugugauuucugaccuauguaacaugguccacuaacucucaguauccaauccguccucgga -3'  exp
    .......((((((.......)))))).((...(((.((((.((((.(((((((..((((((((.(........).).)))))))..))))))).)))).)))))))...))..((((....))))  reads  mm
    ...................cacuccacgguuccugcagaga....................................................................................  1      0
    ....................acuccacgguuccugcagaga....................................................................................  1      0
    .....................cuccacgguuccugcagaga....................................................................................  2      0
    ........................................augguagacuauggaacguag................................................................  2      0
    ........................................augguagacuauggaacguagg...............................................................  8      0
    .........................................ugguagacuauggaacg...................................................................  5      0
    .........................................ugguagacuauggaacgu..................................................................  626    0
    .........................................ugguagacuauggaacgua.................................................................  700    0
    .........................................ugguagacuauggaacguag................................................................  4608   0
    .........................................ugguagacuauggaacguagg...............................................................  33217  0
    .........................................ugguagacuauggaacguaggc..............................................................  5523   0
    .........................................ugguagacuauggaacguaggcu.............................................................  1060   0
    .........................................ugguagacuauggaacguaggcug............................................................  8      0
    .........................................ugguagacuauggaacguaggcugu...........................................................  1      0
    .........................................ugguagacuauggaacguaggcugugu.........................................................  1      0
    ..........................................gguagacuauggaacgua.................................................................  1      0
    ..........................................gguagacuauggaacguag................................................................  5      0
    ..........................................gguagacuauggaacguagg...............................................................  26     0
    ..........................................gguagacuauggaacguaggc..............................................................  5      0
    ...........................................guagacuauggaacguagg...............................................................  1      0
    ..............................................................cugugugauuucugacU..............................................  4      1
    ..............................................................cugugugauuucugacc..............................................  12     0
    ..............................................................................cuauguaacauggucccacu............................  1      0
    ..............................................................................cuauguaacauggucccacua...........................  1      0
    ..............................................................................cuauguaacauggucccacuaa..........................  1      0
    ..............................................................................cuauguaacauggucccacuaac.........................  38     0
    ..............................................................................cuauguaacauggucccacuaacu........................  5      0
    ...............................................................................uauguaacauggucccacA.............................  1      1
    ...............................................................................uauguaacauggucccacu............................  19     0
    ...............................................................................uauguaacauggucccacua...........................  11     0
    ...............................................................................uauguaacauggucccacuaG..........................  3      1
    ...............................................................................uauguaacauggucccacuaa..........................  26     0
    ...............................................................................uauguaacauggucccacuaac.........................  2072   0
    ...............................................................................uauguaacauggucccacuaacu........................  256    0
```

```
                                                                                    oar-miR-379-3p
                                         oar-miR-379-5p
cugauucuggggucagcaccacuccacgguuccugcagagaugguagacuauggaacguaggcugugugauuucugaccuauguaacaugguccacuaacucucaguauccaauccguccucgga

................................................................................auguaacaugguccacCa...........................    1    1
................................................................................auguaacaugguccacuaac.........................    2    0
```
